# Cadmium‐Induced Mitochondrial and MAMs Dysregulation in Rat Testis: The Protective Role of D‐Aspartate

**DOI:** 10.1002/tox.24559

**Published:** 2025-08-04

**Authors:** Debora Latino, Sara Falvo, Massimo Venditti, Alessandra Santillo, Giulia Grillo, Gabriella Chieffi Baccari, Imed Messaoudi, Maria Maddalena Di Fiore

**Affiliations:** ^1^ Department of Environmental, Biological and Pharmaceutical Sciences and Technologies University of Campania “Luigi Vanvitelli” Caserta Italy; ^2^ Department of Experimental Medicine, Section Human Physiology and Integrated Biological Functions University of Campania “Luigi Vanvitelli” Napoli Italy; ^3^ 3LR11ES41: Génetique, Biodiversité et Valorisation des Bioressources Institut Supérieur de Biotechnologie, Université de Monastir Monastir Tunisia

**Keywords:** cadmium, D‐aspartate, MAMs, mitochondria, testis

## Abstract

Cadmium (Cd), a heavy metal, disrupts the structure of seminiferous tubules and induces cell death at multiple stages of sperm development. Cd also impairs Leydig cells (LCs), resulting in reduced serum testosterone (T) levels. This study primarily examined the impact of Cd on the mitochondrial compartment and mitochondrial‐associated endoplasmic reticulum membranes (MAMs) in rat testis. Additionally, the potential of D‐aspartate (D‐Asp) to mitigate Cd‐induced effects on steroidogenesis and spermatogenesis was assessed by administering D‐Asp simultaneously or preventively with Cd. The findings demonstrated that Cd exerts reprotoxicity by affecting the mitochondrial compartment and MAMs, evidenced by an imbalance in mitochondrial dynamics, impaired mitophagy pathway, and downregulated mitochondrial biogenesis. Cd exposure also reduced lipid transfer‐related factor expression and increased ER stress. Moreover, elevated levels of Ca^2+^ transfer‐related proteins, indicative of perturbed Ca^2+^ homeostasis, may be associated with enhanced oxidative stress and apoptosis, which are known effects of Cd. Immunofluorescent analysis revealed that the Cd‐induced mitochondrial and MAMs damage was prominent in LCs, spermatocytes, and spermatids, confirming the metal's adverse effects on steroidogenesis and spermatogenesis. Conversely, co‐administration or preventive administration of D‐Asp with Cd preserved mitochondrial homeostasis and functional ER‐mitochondria interactions. In conclusion, the study offers novel insights into the cellular mechanisms underlying Cd‐induced reprotoxicity. Importantly, it highlights the efficacy of D‐Asp in preventing or counteracting testicular damage caused by Cd by enhancing mitochondrial and MAMs functionality.

**Abbreviations**
ATAD3A: ATPase family AAA domain containing 3A
Ca^2+^
: calcium ions
Cd: cadmium
CdCl_2_
: cadmium chloride
DAPI: 4′,6‐diamidino‐2‐phenylindole
D‐Asp: D‐aspartate
DRP1: dynamin‐related protein 1
ER: endoplasmic reticulum
FACL4: long‐chain‐fatty acid—CoA ligase
GRP75: glucose‐regulated protein 75
GRP78: glucose‐regulated protein 78
IF: immunofluorescence
LCs: Leydig cells
MAMs: mitochondria‐associated ER membranes
MFN1: mitofusin 1
MFN2: mitofusin 2
NRF1: nuclear respiratory factor 1
OPA1: optic atrophy 1
P450scc: side‐chain cleavage enzyme
PINK1: PTEN‐induced kinase 1
SCs: Sertoli cells
S.D.: standard deviation
SDS‐PAGE: sodium dodecyl sulfate–polyacrylamide gel electrophoresis
SOAT1: sterol O‐acyltransferase 1
T: testosterone
TFAM: mitochondrial transcription factor A
TOMM20: translocase of the outer mitochondrial membrane complex subunit 20
VDAC: voltage‐dependent anion channels
WB: Western blot

## 1 Introduction

Heavy metal exposure can significantly impair male fertility by causing various forms of damage, inducing oxidative stress, apoptosis, and autophagy in testicular tissue [1, 2]. Among heavy metals, cadmium (Cd) poses a considerable risk to male reproductive health by impairing the seminiferous tubules, which are critical for sperm cell development, and inducing cell death at various stages of spermatogenesis [3, 4, 5, 6, 7, 8, 9]. Cd also affects Leydig cells (LCs), which produce testosterone (T) and Sertoli cells (SCs), essential for nurturing developing sperm cells [9, 10, 11]. These damages result in reduced T levels, sperm count, motility, and viability, ultimately leading to infertility [12, 13].

Cd's primary cellular target is the mitochondrion, where it induces oxidative stress and generates reactive oxygen species (ROS). These effects activate apoptosis, cause mtDNA mutations, alter gene expression, inhibit respiratory chain complexes, and reduce ATP synthesis in organs such as the liver and kidneys [14]. Mitochondria are crucial for steroidogenesis, as they contain enzymes that initiate the steroidogenesis pathway, which concludes in the endoplasmic reticulum (ER) to produce steroid hormones [15]. Mitochondria and the ER are interconnected via mitochondrial‐associated ER membranes (MAMs), specialized structures that regulate steroidogenesis by facilitating the transfer of cholesterol and steroid intermediates like pregnenolone between these organelles [16]. MAMs also enable the transfer of Ca^2+^ ions, influencing cellular processes, including the ER stress response [17]. Additionally, MAMs play roles in lipid biosynthesis, trafficking, mitochondrial dynamics, and mitophagy [17]. Studies in organs such as the spleen, heart, and neurons under Cd exposure reveal significant reductions in MAMs‐related gene and protein expressions, leading to MAMs dysfunction [18, 19, 20]. However, the effects of Cd on mitochondrial and MAMs functionality in the testis remain unexplored.

Given the ubiquitous presence of Cd as an environmental toxicant, exposure to this metal is nearly unavoidable. Consequently, investigating natural molecules as protective agents against Cd toxicity is critical for mitigating its associated risks. Recent studies have highlighted the protective role of D‐aspartate (D‐Asp) in countering Cd‐induced toxicity in rat testes [11]. D‐Asp mitigates oxidative stress, promotes cell survival, protects testicular cells from apoptosis, and supports normal spermatogenesis and steroidogenesis [11]. It is well recognized as a key regulator of steroidogenesis, particularly within the testes, where it facilitates molecular cascades leading to enhanced T production [21, 22, 23, 24]. Evidence from Ddo knockin mice, which exhibit depleted D‐Asp levels, further confirms its critical role, as these animals experience alterations in both steroidogenesis and spermatogenesis [25].

Recent findings also demonstrate that D‐Asp improves the steroidogenic process in rat testes by enhancing mitochondrial functionality and its interaction with the ER via MAMs [26]. This study aimed to investigate the impact of Cd on the mitochondrial compartment and MAMs in rat testes. Additionally, considering the established potential of D‐Asp in alleviating Cd‐induced adverse effects on steroidogenesis and spermatogenesis, experiments were conducted wherein rats were treated with D‐Asp either simultaneously with or preventively to Cd exposure.

## 2 Material and Methods

### 2.1 Rats and Experimental Design

Thirty 60‐day‐old male Wistar rats were used in this study. They were housed individually under controlled conditions, maintaining a 12:12 light/dark cycle, a temperature of 22°C ± 2°C, and humidity at 55% ± 20%. The animals were provided with a standard rodent diet and had ad libitum access to water. The rats were divided into six groups, each consisting of five animals: Control groups (C 15 and C 30) were given 2 mL distilled water daily via gastric gavage per rat for 15 or 30 days; the D‐Asp group (D‐Asp) received a daily dose of 0.1 mM D‐Asp per gram of body weight through gastric gavage for 15 days (D‐Asp; 219096; Sigma‐Aldrich, Milan, Italy); the Cd group (Cd) was administered 5 μg Cd per day per gram of body weight for 15 days via gastric gavage (CdCl_2_; Sigma‐Aldrich, Milan, Italy); the Cd+D‐Asp group (Cd+D‐Asp) was treated with Cd in the morning followed by D‐Asp in the afternoon for 15 days; and the D‐Asp/Cd group (D‐Asp/Cd) received D‐Asp for 15 days followed by Cd for the subsequent 15 days. Body weight measurements were taken every 5 days. The chosen doses and administration methods for both Cd and D‐Asp were based on prior research [21, 27].

### 2.2 Sample Collection

Following the experimental treatments, all animals were euthanized with intraperitoneal injection of 4% chloral hydrate (10 mL/kg). Testes were excised from each rat and weighed. One testis was fixed in 10% neutral buffered formalin for IF analysis, while the other was stored at −80°C for molecular studies.

### 2.3 Protein Extraction and Western Blot (WB) Analysis

Testes were homogenized in RIPA lysis buffer (#TCL131; Hi Media Laboratories GmbH; Einhausen, Germany) supplemented with 10 μL/mL of a protease inhibitor mix (#39102; SERVA Electrophoresis GmbH; Heidelberg, Germany). Homogenates were centrifuged at 14000 ×g for 20 min at 4°C. Protein concentrations were measured using the Bradford assay (Bio‐Rad). Thirty micrograms of total protein extracts were separated on 12% polyacrylamide SDS‐PAGE and processed as described by [9]. Details of all primary and secondary antibodies used are provided in Table 1. Protein quantification was performed using ImageJ software (version 1.53t; National Institutes of Health, Bethesda, USA). Each WB analysis was conducted in triplicate.

**TABLE 1:** List of all the used antibodies.

Antibody	Molecular weight (kDa)	WB dilution	IF dilution	Source
ATAD3A	70	1:1000	1:50	Sigma‐Aldrich, Saint Louis, MO, USA SAB3500668
DRP1	78–82	1:1000	—	Cell Signaling Technology, Danvers, Ma, USA #8570
FACL4	79	1:1000	—	Abcam, Cambridge, United Kingdom #ab227256
GRP75	75	1:1000	—	Cell Signaling Technology, Danvers, Ma, USA #2816
GRP78	78	1:1000	1:100	Cell Signaling Technology, Danvers, Ma, USA #3183
MFN1	84	1:1000	1:100	Abcam, Cambridge, UK #ab221661
MFN2	86	1:500	1:100	Abcam, Cambridge, UK #ab124773
NRF1	68	1:1000	—	Cell Signaling Technology, Danvers, Ma, USA #69432
OPA1	93–111	1:1000	—	Abcam, Cambridge, UK #ab42364
PARKIN	52	1:1000	—	Abcam, Cambridge, UK #AB77924
PINK1	63	1:1000	1:100	Abcam, Cambridge, UK #ab216144
SOAT1/ACAT1	47	1:1000	—	Abcam, Cambridge, UK #ab39327
TFAM	28	1:2000	1:100	Abcam, Cambridge, United Kingdom #ab131607
TOMM20	16	1:1000	1:100	Sigma‐Aldrich, Milan, Italy WH0009804M1
VDAC	32	1:1000	1:100	Cell Signaling Technology, Danvers, Ma, USA #4661
β‐actin	42	1:2000	1:100	Elabscience Biotechnology, Wuhan, China #E‐AB‐20031
Goat anti‐mouse CF 568	—	—	1:200	Sigma‐Aldrich, Saint Louis, MO, USA #SAB4600082
Goat anti‐mouse IgG HRP	—	1:2000	—	BioActs, Namdong‐gu, Incheon, Korea #RSA1122
Goat anti‐rabbit Alexa Fluor 488	—	—	1:500	Thermo Fisher Scientific, Waltham, Ma, USA #A32731
Goat anti‐rabbit IgG HRP	—	1:3000	—	Vector Laboratories, Burlingame, CA, USA #PI‐1000
PNA lectin Alexa Fluor 568	—		1:50	Thermo Fisher Scientific, Waltham, Ma, USA #L32458

### 2.4 Immunofluorescence Analysis

For IF staining, 5‐μm testis sections were dewaxed, rehydrated, and processed according to the method described by Venditti et al. [9]. Details of the antibodies used are provided in Table 1. Cell nuclei were stained with Vectashield containing DAPI (Vector Laboratories, Peterborough, UK) and observed under an optical microscope (Leica DM 5000 B + CTR 5000; Leica Microsystems, Wetzlar, Germany) equipped with a UV lamp. Images were captured using the Leica DFC320 R2 digital camera, analyzed, and saved using IM 1000 software (version 4.7.0; Leica Microsystems, Wetzlar, Germany). Densitometric analysis of IF signals was conducted using the Fiji plugin (version 3.9.0/1.53t) of ImageJ software. A total of 20 seminiferous tubules per animal were analyzed, amounting to 100 tubules per group. All IF analyses were conducted in triplicate.

### 2.5 Statistical Analysis

Analysis of variance (ANOVA) followed by the Student–Newman–Keuls test was applied to assess significant differences among experimental groups. Statistical significance was considered at *p* < 0.05. All results were expressed as mean ± standard deviation (S.D.).

## 3 Results

This study analyzed the expression of key proteins associated with mitochondrial homeostasis and dynamics, including those involved in fusion, fission, biogenesis, mitophagy, and mitochondrial mass. Additionally, interactions between the ER and mitochondria, particularly those related to lipid transfer and Ca^2+^ signaling, as well as markers of ER stress, were investigated.

To assess the potential protective role of D‐Asp against Cd‐induced damage, one group of rats was co‐treated with D‐Asp and Cd for 15 days (Cd+D‐Asp group), while another group received D‐Asp for 15 days followed by Cd for an additional 15 days (D‐Asp/Cd group). Since the treatment period for the D‐Asp/Cd group spanned 30 days, a second control group was included, consisting of rats housed for 30 days. No significant differences were observed between the two control groups (C15 and C30) across all parameters; therefore, their mean values were used for graphical representation of WB and IF analyses, collectively referred to as controls (C).

### 3.1 Effects of Cd and/or D‐Asp on Mitochondrial Compartment

#### 3.1.1 Effects of Cd and/or D‐Asp on Fusion and Fission

**FIGURE 1:**
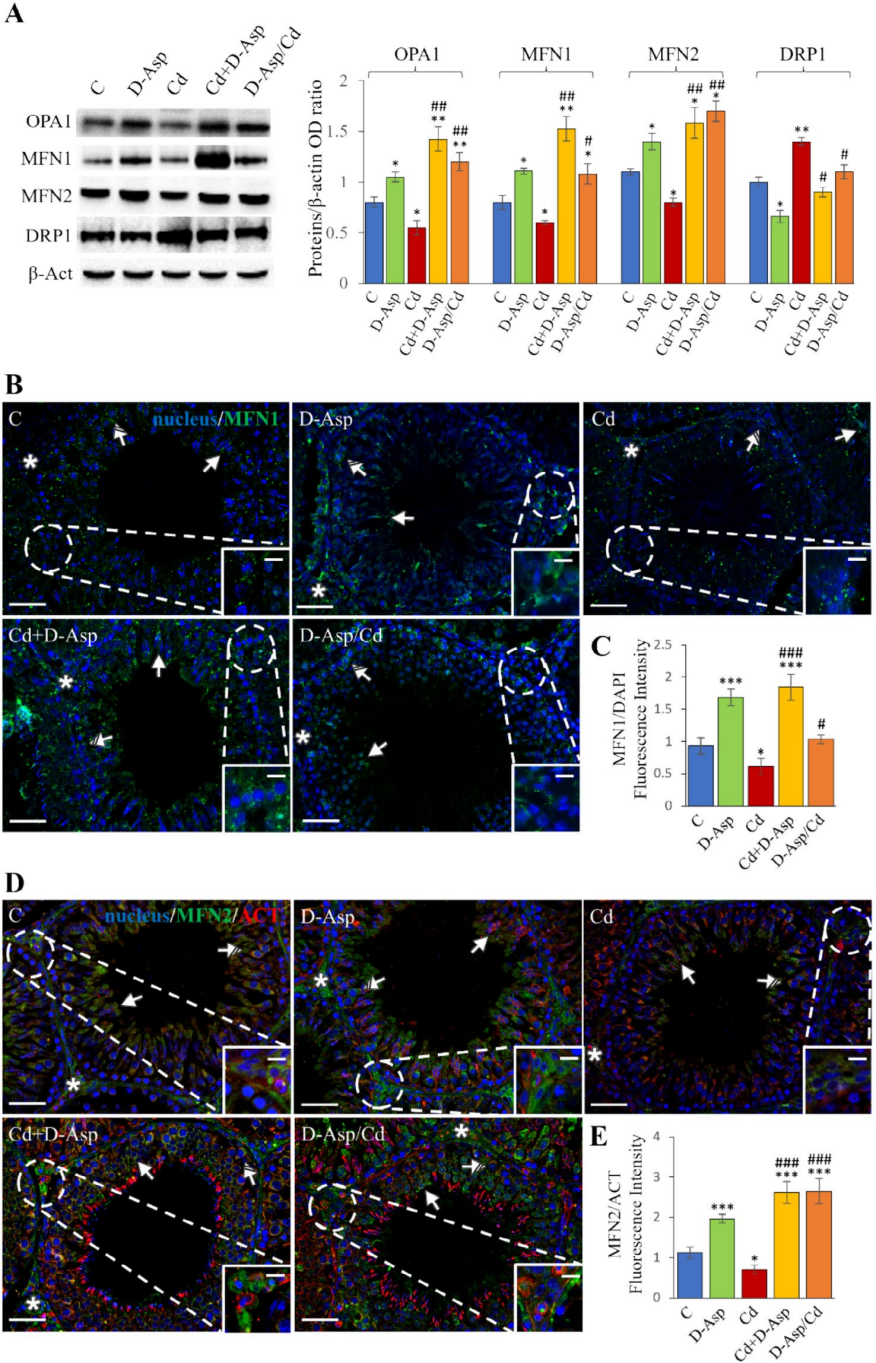
Analysis of mitochondrial fusion and fission in C, D‐Asp‐, Cd‐, Cd+D‐Asp‐, and D‐Asp/Cd‐treated rats. (A) Protein expression levels of OPA1 (93–111 kDa), MFN1 (84 kDa), MFN2 (86 kDa), and DRP1 (78–82 kDa) by Western blot in D‐Asp‐ and/or Cd‐rat testes. The expression levels of OPA1, MFN1, MFN2, and DRP1 were quantified using ImageJ and normalized to β‐actin (42 kDa). (B) Immunolocalization of MFN1 in D‐Asp‐ and/or Cd‐rat testes (green). (D) Immunolocalization of MFN2 (green) and β‐actin (red) in D‐Asp‐ and/or Cd‐rat testes. All slides were counterstained with DAPI (blue) and captured at ×20. Scale bars 20 μm; in the insets 10 μm. Striped arrows: Spermatocytes; arrows: Spermatids; asterisks: LCs. The histograms show the quantification of MFN1 (C) and MFN2 (E) fluorescence signal intensity. All values are expressed as mean ± SD from five animals in each group. *, D‐Asp, Cd, Cd+D‐Asp, D‐Asp/Cd versus Control: *p < 0.05; **p < 0.01; ***p < 0.001; #, Cd+D‐Asp, D‐Asp/Cd versus Cd: #*p* < 0.05; ##*p* < 0.01; ###*p* < 0.001.

The expression levels of OPA1, MFN1, and MFN2 were analyzed to evaluate the impact of treatments on mitochondrial fusion. WB results indicated that D‐Asp treatment significantly increased OPA1, MFN1, and MFN2 levels compared to controls (*p* < 0.05). Conversely, Cd treatment significantly reduced their expression levels (*p* < 0.05) (Figure 1A). In the Cd+D‐Asp group, OPA1, MFN1, and MFN2 expression levels were significantly elevated compared to controls (*p <* 0.01 for OPA1 and MFN1; *p <* 0.05 for MFN2) and Cd‐treated rats (*p* < 0.01) (Figure 1A). Notably, pre‐treatment with D‐Asp for 15 days fully prevented Cd‐induced reductions in these proteins, with expression levels exceeding those of controls (*p* < 0.01 for OPA1; p < 0.05 for MFN1 and MFN2) (Figure 1A).

The effects of all treatments on mitochondrial fusion were further validated through IF staining of MFN1 and MFN2, as shown in Figure 1B,D, respectively. In the testes of control (C) and D‐Asp‐treated groups, MFN1 was predominantly localized in the cytoplasm of germ cells, specifically in spermatocytes (striped arrows) and round spermatids (arrows), with positive staining also evident in interstitial LCs (asterisks; insets) (Figure 1B). In the Cd‐treated group, a significant reduction in MFN1 staining was observed in both germ cells and LCs compared to controls (*p* < 0.05). Conversely, a substantial increase in the fluorescent signal was detected in the Cd+D‐Asp group compared to both C (*p* < 0.001) and Cd groups (*p* < 0.001). In the D‐Asp/Cd group, MFN1 signal intensity was comparable to controls, confirming the protective role of D‐Asp in counteracting Cd‐induced damage (Figure 1C). Regarding MFN2, its localization was observed in spermatocytes (striped arrows), where co‐localization with β‐actin produced a yellow‐orange tint, in spermatids (arrows), and prominently in interstitial LCs (asterisks; inset) in the C and D‐Asp groups (Figure 1D). In the Cd‐treated group, a pronounced reduction in MFN2 signal intensity was observed compared to controls (*p* < 0.05) (Figure 1E). In contrast, both the Cd+D‐Asp and D‐Asp/Cd groups displayed significantly increased MFN2 fluorescent signal intensity relative to the C and Cd groups (*p* < 0.001) (Figure 1E).

The effect of Cd and/or D‐Asp on DRP1, a key regulator of mitochondrial fission, was also evaluated. DRP1 expression was lower in the D‐Asp‐treated group compared to controls (*p* < 0.05), suggesting that D‐Asp promotes a shift in mitochondrial dynamics toward fusion. Cd treatment, however, caused a significant increase in DRP1 expression compared to controls (*p* < 0.01) (Figure 1A). Co‐treatment with D‐Asp or pre‐treatment for 15 days significantly decreased DRP1 levels relative to the Cd‐treated group (*p* < 0.05) (Figure 1A).

#### 3.1.2 Effects of Cd and/or D‐Asp on Mitochondrial Biogenesis

**FIGURE 2:**
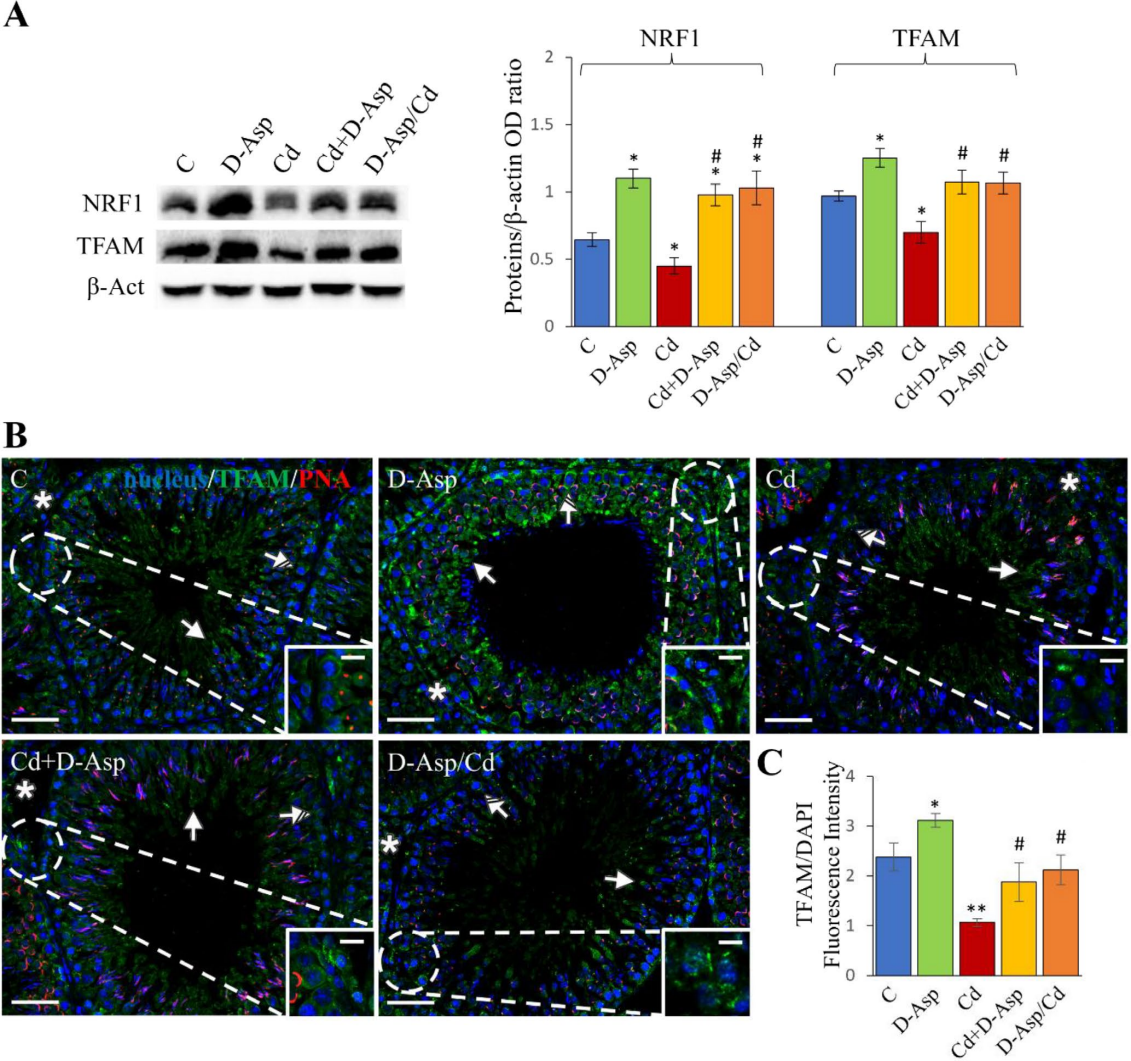
Analysis of mitochondrial biogenesis in C, D‐Asp‐, Cd‐, Cd+D‐Asp‐, and D‐Asp/Cd‐treated rats. (A) Protein expression levels of NRF1 (68 kDa) and TFAM (28 kDa) by Western blot in D‐Asp‐ and/or Cd‐rat testes. The expression levels of NRF1 and TFAM were quantified using ImageJ and normalized to β‐actin (42 kDa). (B) Immunolocalization of TFAM (green) and PNA lectin (red) in D‐Asp‐ and/or Cd‐rat testes. All slides were counterstained with DAPI (blue) and captured at ×20. Scale bars 20 μm; in the insets 10 μm. Striped arrows: Spermatocytes; arrows: Spermatids; asterisks: Leydig cells. (C) The histogram shows the quantification of TFAM fluorescence signal intensity. All values are expressed as mean ± SD from five animals in each group. *, D‐Asp, Cd, Cd+D‐Asp, D‐Asp/Cd versus Control: *p < 0.05; **p < 0.01; #, Cd+D‐Asp, D‐Asp/Cd versus Cd: #*p* < 0.05.

The expression levels of NRF1 and TFAM, two critical markers of mitochondrial biogenesis, were significantly elevated in the D‐Asp‐treated group compared to controls (C) (*p* < 0.05). In contrast, Cd treatment caused a marked reduction in their expression relative to the C group (*p* < 0.05) (Figure 2A). Notably, in both the Cd+D‐Asp and D‐Asp/Cd groups, NRF1 expression levels were significantly increased compared to both the C and Cd‐treated groups (*p* < 0.05). TFAM expression levels were fully restored in these groups, matching those of the controls and exceeding the levels observed in the Cd group (*p* < 0.05) (Figure 2A).

To confirm these findings, IF staining of TFAM was conducted (Figure 2B). In control and D‐Asp‐treated testes, the TFAM signal was prominently localized in LCs (asterisks; inset), spermatocytes (striped arrows), and elongating spermatids (arrows). The fluorescence signal intensity was stronger in the D‐Asp group compared to the C group (*p* < 0.05; Figure 2C). Conversely, a significant reduction in TFAM fluorescence intensity was observed in the Cd‐treated group within the same cell types, as compared to the C group (*p* < 0.01). Importantly, no differences in TFAM fluorescence intensity were detected among the Cd+D‐Asp, D‐Asp/Cd, and control groups, highlighting the ability of D‐Asp to mitigate Cd‐induced reductions in mitochondrial biogenesis markers.

#### 3.1.3 Effects of Cd and/or D‐Asp on Mitophagy

Mitophagy, a selective autophagy pathway for removing defective mitochondria, is regulated by the coordinated action of PINK1 and PARKIN proteins. Upon mitochondrial damage, PINK1 accumulates on the outer mitochondrial membrane, recruiting PARKIN to facilitate autophagosome assembly and degradation of the damaged mitochondria.

**FIGURE 3:**
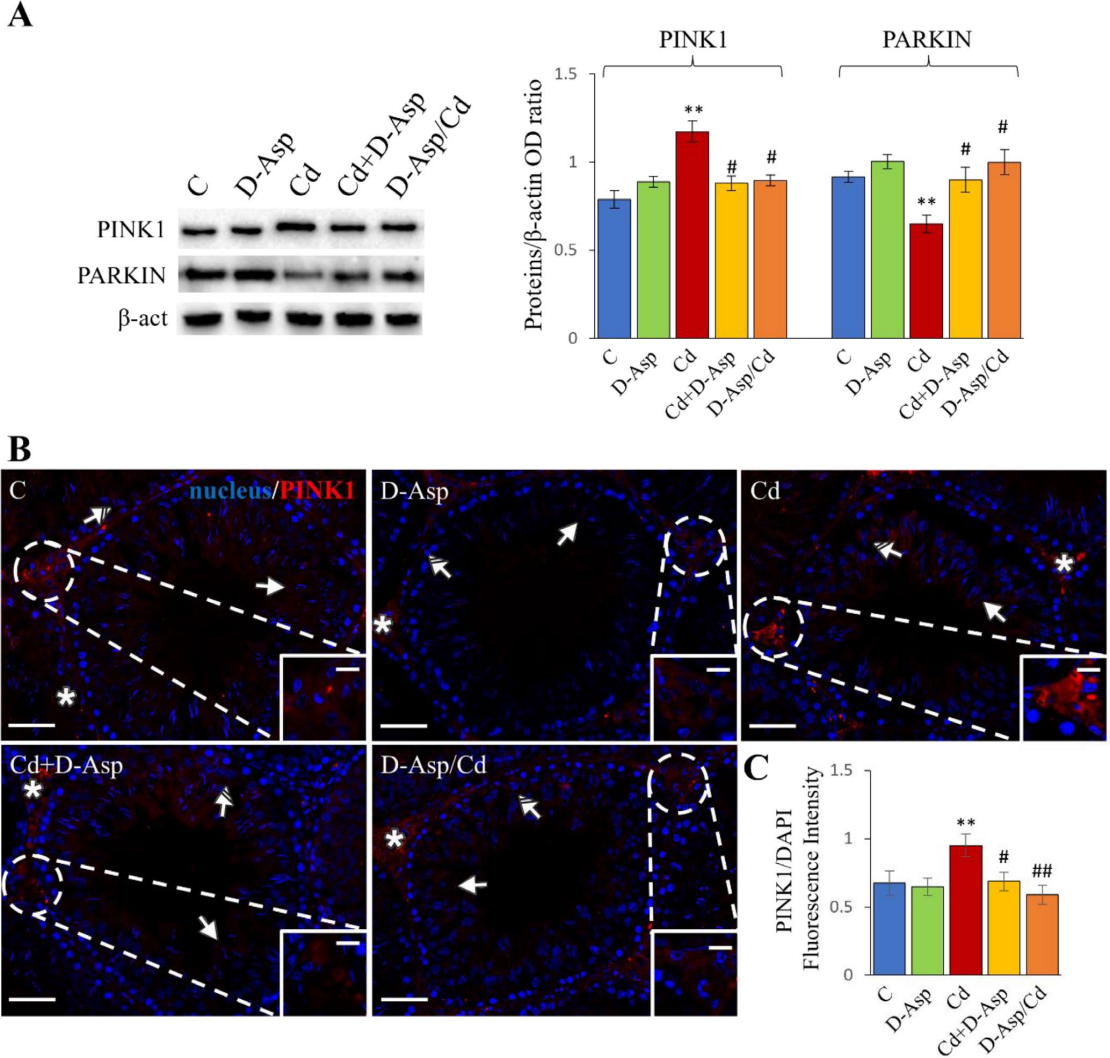
Analysis of mitophagy in C, D‐Asp‐, Cd‐, Cd+D‐Asp‐, and D‐Asp/Cd‐treated rats. (A) Protein expression levels of PINK1 (63 kDa) and PARKIN (52 kDa) by Western blot in D‐Asp‐ and/or Cd‐rat testes. The expression levels of PINK1 and PARKIN were quantified using ImageJ and normalized to β‐actin (42 kDa). (B) Immunolocalization of PINK1 (green) in D‐Asp‐ and/or Cd‐rat testes. All slides were counterstained with DAPI (blue) and captured at ×20. Scale bars 20 μm; in the insets 10 μm. Striped arrows: Spermatocytes; arrows: Spermatids; asterisks: Leydig cells. (C) The histogram shows the quantification of PINK1 fluorescence signal intensity. All values are expressed as mean ± SD from five animals in each group. *, D‐Asp, Cd, Cd+D‐Asp, D‐Asp/Cd versus Control: *p < 0.05; **p < 0.01; #, Cd+D‐Asp, D‐Asp/Cd versus Cd: #*p* < 0.05; ##*p* < 0.01.

In this study, PINK1 protein levels were significantly elevated in the testes of Cd‐exposed rats compared to the C group (*p* < 0.01) (Figure 3A). However, D‐Asp treatment, whether administered simultaneously with Cd or as a 15‐day pre‐treatment, significantly reduced PINK1 levels relative to the Cd group (*p* < 0.05), restoring levels comparable to those of the C group (Figure 3A). Conversely, PARKIN expression levels were markedly reduced in the Cd group compared to the C group (*p* < 0.01). In the D‐Asp, Cd+D‐Asp, and D‐Asp/Cd groups, PARKIN levels were similar to those in the controls, showing no significant differences (Figure 3A). These findings suggest that Cd disrupts the mitophagy pathway by increasing PINK1 accumulation while reducing PARKIN expression. D‐Asp administration, whether alone or in combination with Cd, effectively maintained the functionality of the mitophagy pathway, mitigating the Cd‐induced alterations.

The effects of all treatments on mitophagy were further examined through PINK1‐IF staining (Figure 3B). In control (C), D‐Asp, Cd+D‐Asp, and D‐Asp/Cd groups, PINK1 was faintly localized in the cytoplasm of spermatocytes (striped arrows), round spermatids (solid arrows), and LCs (asterisks; insets). No significant differences in fluorescent intensity were detected among these groups (Figure 3B,C). However, in the Cd‐treated group, PINK1 signal intensity was significantly elevated, especially in LCs, compared to controls (*p* < 0.01) (Figure 3C). In both the Cd+D‐Asp and D‐Asp/Cd groups, PINK1 fluorescence intensity was significantly reduced compared to the Cd group (*p* < 0.05 and *p* < 0.01, respectively), returning to levels comparable to the controls (Figure 3C).

#### 3.1.4 Effects of Cd and/or D‐Asp on Mitochondrial Mass

**FIGURE 4:**
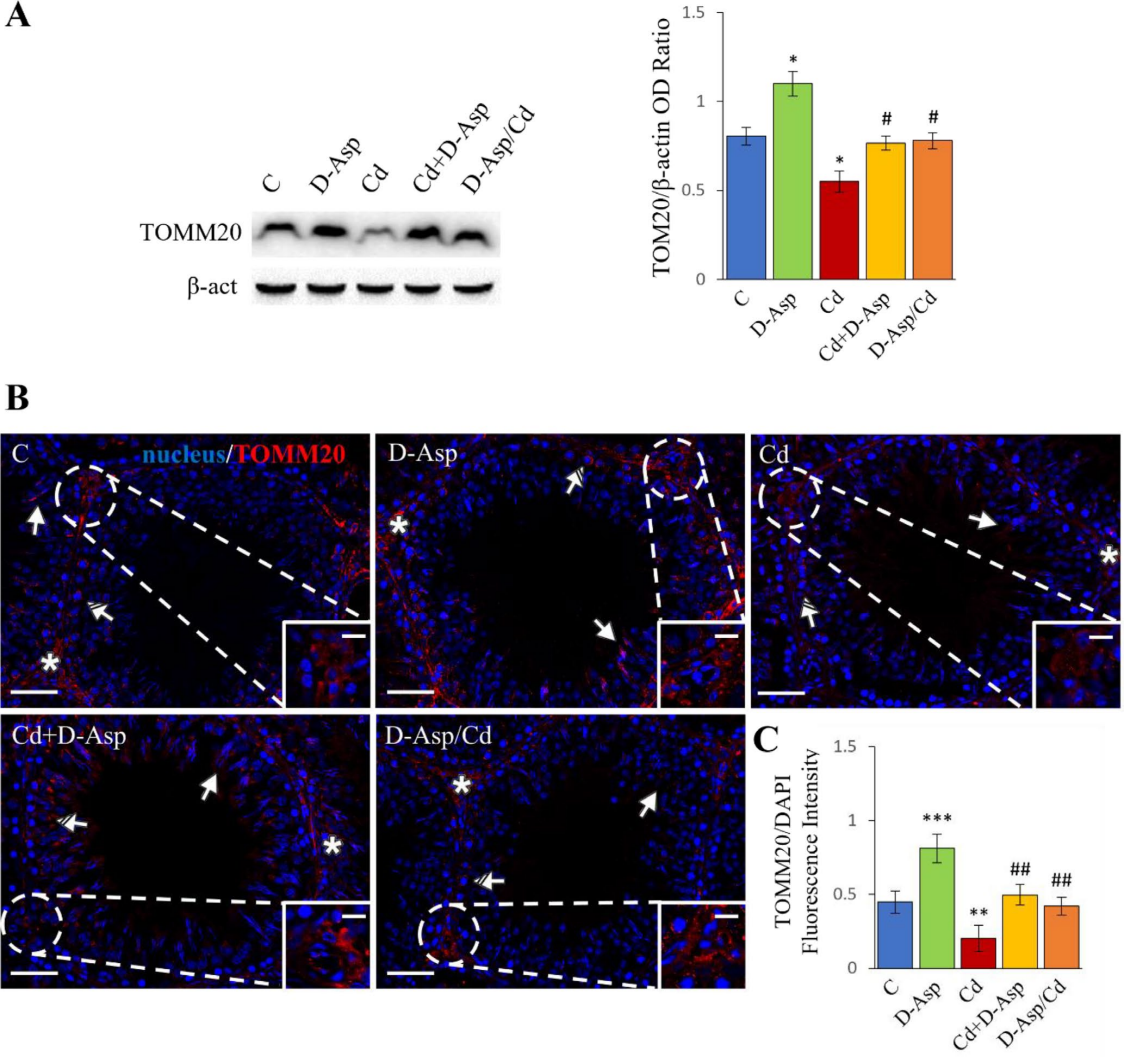
Mitochondrial mass analysis in C, D‐Asp‐, Cd‐, Cd+D‐Asp‐, and D‐Asp/Cd‐treated rats. (A) Protein expression levels of TOMM20 (16 kDa) by Western blot in D‐Asp‐ and/or Cd‐rat testes. The expression levels of TOMM20 were quantified using ImageJ and normalized to β‐actin (42 kDa). (B) Immunolocalization of TOMM20 (red) in D‐Asp‐ and/or Cd‐rat testes. All slides were counterstained with DAPI (blue) and captured at ×20. Scale bars 20 μm; in the insets 10 μm. Striped arrows: Spermatocytes; arrows: Spermatids; asterisks: Leydig cells. (C) The histogram shows the quantification of TOMM20 fluorescence signal intensity. All values are expressed as mean ± SD from five animals in each group. *, D‐Asp, Cd, Cd+D‐Asp, D‐Asp/Cd versus Control: *p < 0.05; **p < 0.01; ***p < 0.001; #, Cd+D‐Asp, D‐Asp/Cd versus Cd: #*p* < 0.05; ##*p* < 0.01.

The effects of D‐Asp and Cd, administered alone or in combination, on mitochondrial mass were assessed by analyzing the protein expression levels of TOMM20, a marker of mitochondrial mass and a component of the outer mitochondrial membrane translocase receptor system. WB analysis revealed that TOMM20 levels were significantly higher in the D‐Asp group compared to controls (*p* < 0.05), while Cd exposure caused a marked reduction in TOMM20 expression levels (*p* < 0.05) (Figure 4A). Co‐treatment with D‐Asp and Cd, as well as D‐Asp pre‐treatment, fully restored TOMM20 expression levels to control values (Figure 4A).

To further investigate TOMM20 localization in rat testes, IF staining was performed (Figure 4B). In the control and D‐Asp groups, TOMM20 was predominantly localized in the cytoplasm of spermatocytes (striped arrows), spermatids (solid arrows), and interstitial LCs (asterisks; insets). In the D‐Asp‐treated group, TOMM20 staining intensity was significantly elevated in germ cells and LCs compared to controls (*p* < 0.001), whereas a significant reduction was observed in the Cd group (*p* < 0.01) (Figure 4B,C). Consistent with WB results, D‐Asp administration, either concurrent with or prior to Cd treatment, completely restored TOMM20 expression and localization in the testis (Figure 4C).

### 3.2 Effects of Cd and/or D‐Asp on MAMs

#### 3.2.1 Effects of Cd and/or D‐Asp on Lipid Transfer‐Related Factors

**FIGURE 5:**
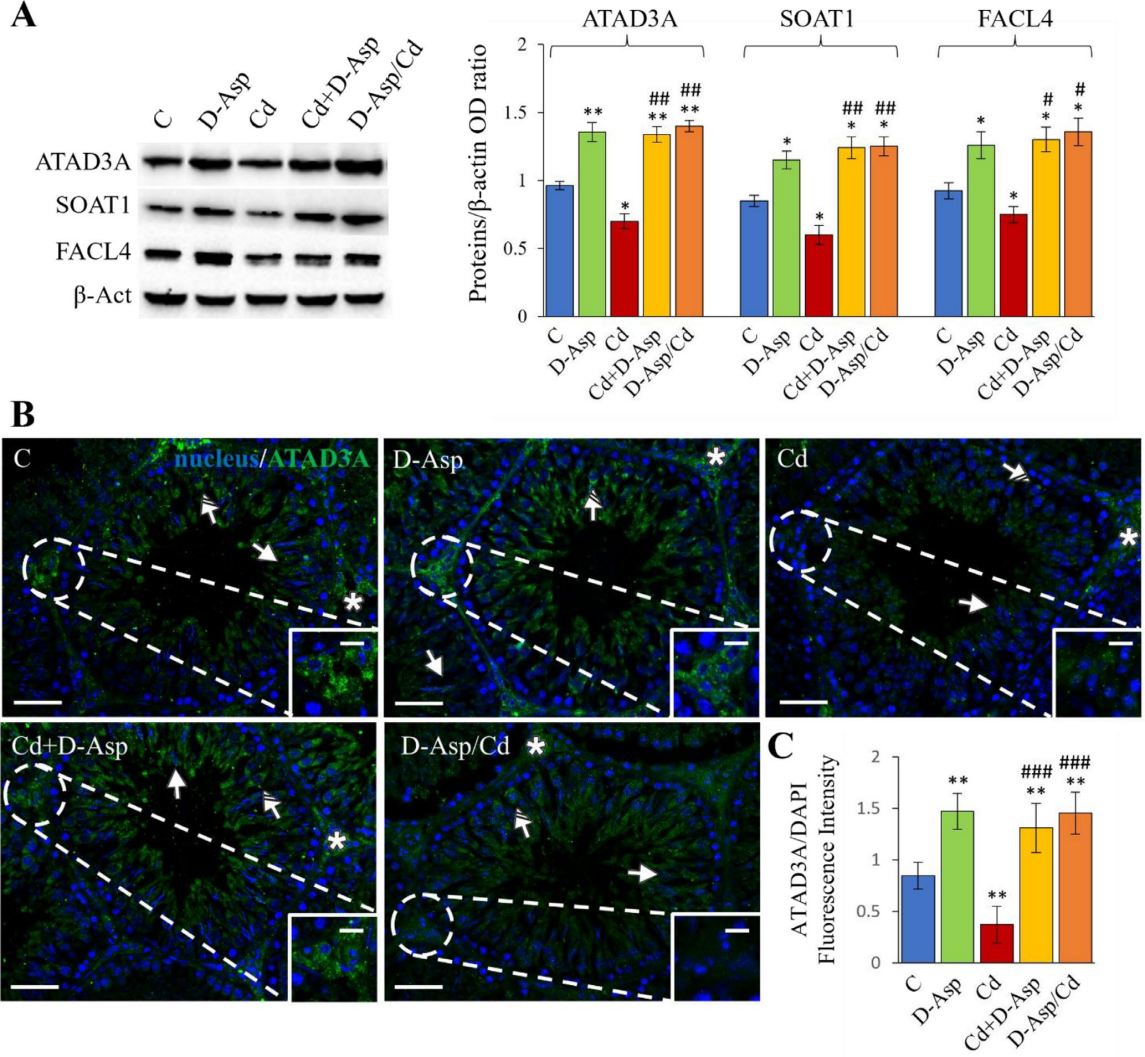
Lipid transfer analysis in C, D‐Asp‐, Cd‐, Cd+D‐Asp‐, and D‐Asp/Cd‐treated rats. (A) Protein expression levels of ATAD3A (70 kDa), (B) SOAT1/ACAT1 (47 kDa), and FACL4 (79 kDa) by Western blot in D‐Asp‐ and/or Cd‐rat testes. The expression levels of ATAD3A, SOAT1, and FACL4 were quantified using ImageJ and normalized to β‐actin (42 kDa). (B) Immunolocalization of ATAD3A (green) in D‐Asp‐ and/or Cd‐rat testes. All slides were counterstained with DAPI (blue) and captured at ×20. Scale bars 20 μm; in the insets 10 μm. Striped arrows: Spermatocytes; arrows: Spermatids; asterisks: Leydig cells. (C) The histogram shows the quantification of ATAD3A fluorescence signal intensity. All values are expressed as mean ± SD from five animals in each group. *, D‐Asp, Cd, Cd+D‐Asp, D‐Asp/Cd versus Control: *p < 0.05; **p < 0.01; #, Cd+D‐Asp, D‐Asp/Cd versus Cd: #*p* < 0.05; ##*p* < 0.01.

The transfer of lipids between the ER and mitochondria is a crucial step in steroidogenesis. To assess this process, the expression levels of ATAD3A, involved in transporting cholesterol from the ER to mitochondria; SOAT1/ACAT1, which converts free cholesterol to cholesterol esters; and FACL4, essential for phospholipid synthesis, were analyzed using WB (Figure 5A). D‐Asp treatment for 15 days significantly increased ATAD3A (*p* < 0.01), SOAT1 (*p* < 0.05), and FACL4 (p < 0.05) protein levels compared to controls (C). In contrast, Cd treatment caused a notable reduction in their expression levels (*p* < 0.05) (Figure 5A). In both Cd+D‐Asp and D‐Asp/Cd groups, the expression levels of these proteins were significantly higher than in the Cd group (*p* < 0.01 for ATAD3A and SOAT1; *p* < 0.05 for FACL4) and the C group (*p* < 0.01 for ATAD3A; *p* < 0.05 for SOAT1 and FACL4), reaching values comparable to those observed in the D‐Asp group (Figure 5A).

Immunofluorescence (IF) staining of ATAD3A further corroborated these findings (Figure 5B). In control and D‐Asp‐treated testes, ATAD3A signal was prominently localized in interstitial LCs (asterisks; inset), the perinuclear space of spermatocytes (striped arrows), and the cytoplasm of elongating spermatids (arrows). The fluorescence intensity was significantly higher in the D‐Asp group compared to controls (*p* < 0.01; Figure 5C). In contrast, Cd‐treated rats displayed a marked decrease in ATAD3A fluorescence intensity in these cell types compared to the control group (*p* < 0.01) (Figure 5C). Interestingly, in the Cd+D‐Asp and D‐Asp/Cd groups, ATAD3A fluorescence intensity was significantly greater than in the Cd group (*p* < 0.001) and the control group (*p* < 0.01), aligning with the levels observed in the D‐Asp group (Figure 5B,C).

#### 3.2.2 Effects of Cd and/or D‐Asp on Ca^2+^ Signaling‐Related Factors

**FIGURE 6:**
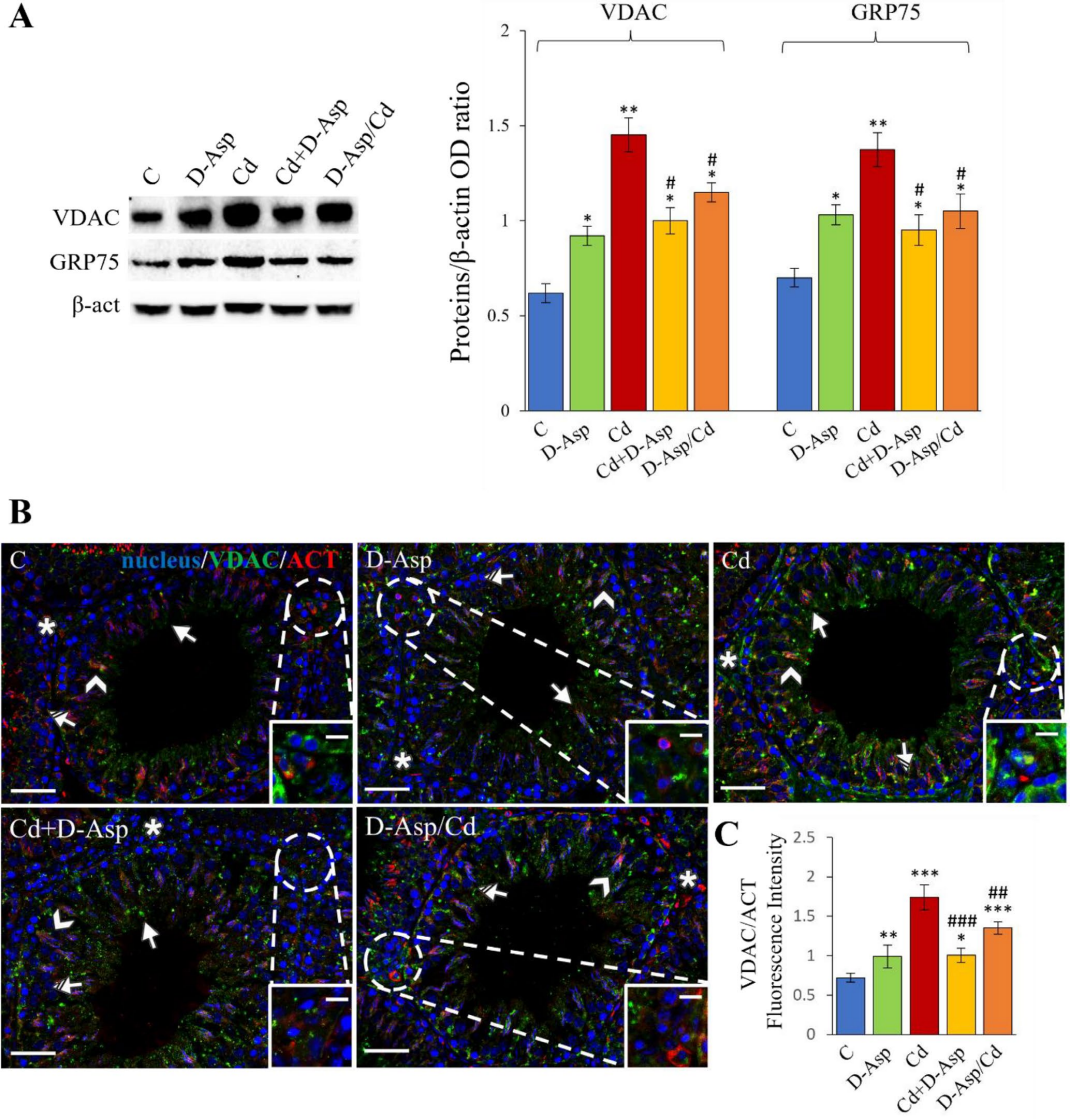
Analysis of calcium signaling in C, D‐Asp‐, Cd‐, Cd+D‐Asp‐, and D‐Asp/Cd‐treated rats. Protein expression levels of (A) VDAC (32 kDa) and GRP75 (75 kDa) by Western blot in D‐Asp‐ and/or Cd‐rat testes. The expression levels of VDAC and GRP75 were quantified using ImageJ and normalized to β‐actin (42 kDa). (B) Immunolocalization of VDAC (green) and β‐actin (red) in D‐Asp‐ and/or Cd‐rat testes. All slides were counterstained with DAPI (blue) and captured at ×20. Scale bars 20 μm; in the insets 10 μm. Striped arrows: Spermatocytes; arrows: Spermatids; arrowheads: Sertoli cells; asterisks: Leydig cells. (C) The histogram shows the quantification of VDAC fluorescence signal intensity. All values are expressed as mean ± SD from five animals in each group. *, D‐Asp, Cd, Cd+D‐Asp, D‐Asp/Cd versus Control: *p < 0.05; **p < 0.01; #, Cd+D‐Asp, D‐Asp/Cd versus Cd: #*p* < 0.05.

The Ca^2+^ signaling pathway, which involves transfer between the ER and mitochondria, plays a pivotal role in steroidogenesis and spermatogenesis progression. Protein levels of VDAC and GRP75, two primary markers of this pathway, were evaluated. In the D‐Asp‐treated group, VDAC and GRP75 expression levels were significantly higher than in controls (C) (*p* < 0.05), while the Cd‐treated group exhibited a marked increase compared to C (*p* < 0.01) (Figure 6A). In the Cd+D‐Asp and D‐Asp/Cd groups, the expression levels of these proteins were similar to those observed in the D‐Asp group (Figure 6A).

To validate these findings, an IF analysis of VDAC co‐localized with β‐actin was conducted. In the control testes, VDAC was localized within both germinal and somatic compartments of seminiferous tubules, specifically in spermatocytes (striped arrow), spermatids (solid arrow), SCs cytoplasmic protrusions (arrowheads), and LCs (asterisks; insets) (Figure 6B). This localization was retained across all treatment groups. However, in the Cd group, VDAC expression was significantly elevated compared to C (*p* < 0.001) (Figure 6C). Interestingly, VDAC fluorescent intensity in the Cd+D‐Asp and D‐Asp/Cd groups was higher than in the C group (*p* < 0.05 and *p* < 0.001, respectively) but lower than in the Cd group (*p* < 0.001 and *p* < 0.01, respectively) (Figure 6C).

#### 3.2.3 Effects of Cd and/or D‐Asp on ER Stress‐Related Factor

**FIGURE 7:**
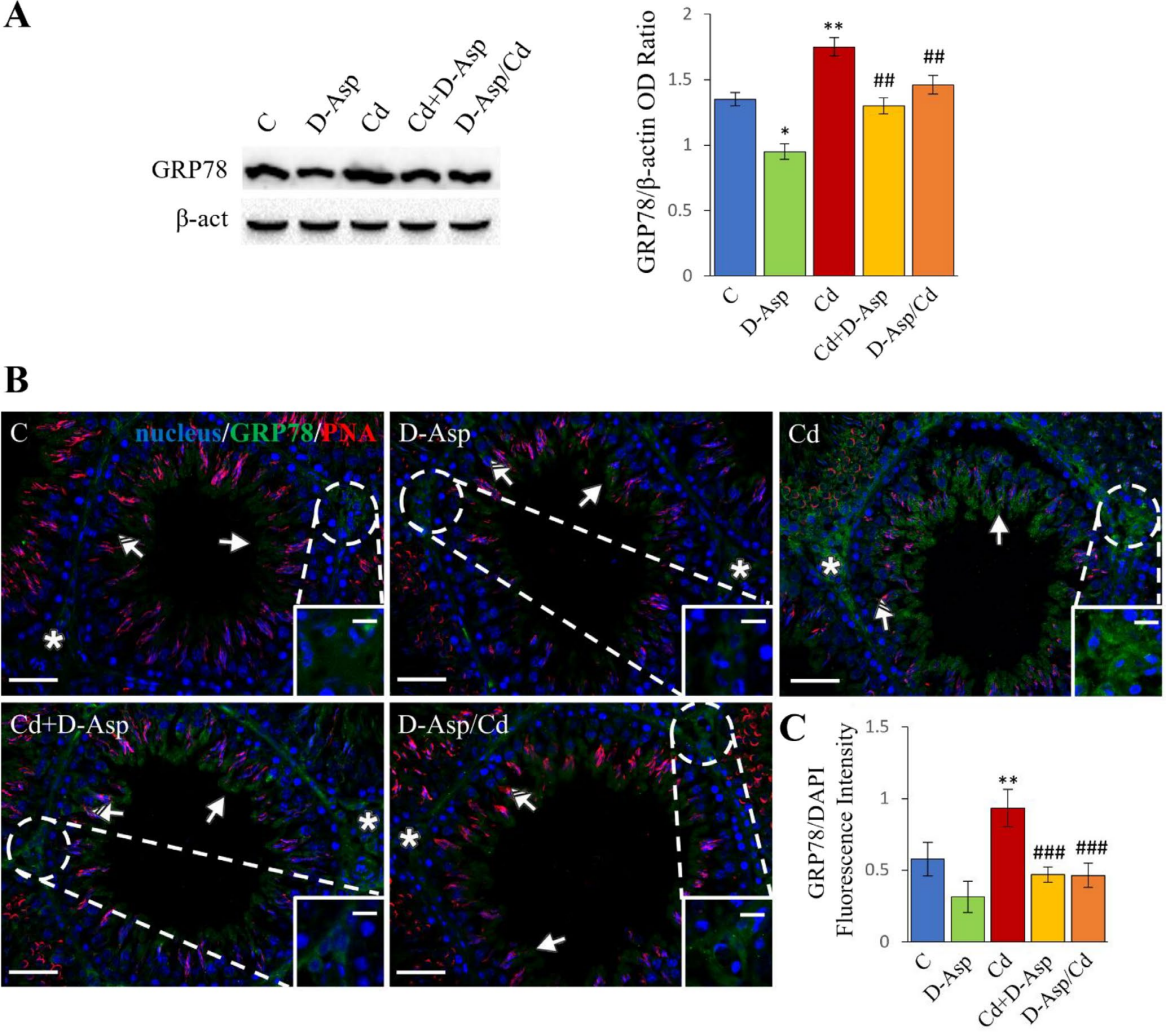
Analysis of ER stress in C, D‐Asp‐, Cd‐, Cd+D‐Asp‐, and D‐Asp/Cd‐treated rats. (A) Protein expression levels of GRP78 (78 kDa) by Western blot in D‐Asp‐ and/or Cd‐rat testes. The expression levels of GRP78 were quantified using ImageJ and normalized to β‐actin (42 kDa). (B) Immunolocalization of GRP78 (green) and PNA lectin (red) in D‐Asp‐ and/or Cd‐rat testes. All slides were counterstained with DAPI (blue) and captured at ×20. Scale bars 20 μm; in the insets 10 μm. Striped arrows: Spermatocytes; arrows: Spermatids; asterisks: Leydig cells. (C) The histogram shows the quantification of GRP78 fluorescence signal intensity. All values are expressed as mean ± SD from five animals in each group. *, D‐Asp, Cd, Cd+D‐Asp, D‐Asp/Cd versus Control: *p < 0.05; **p < 0.01; #, Cd+D‐Asp, D‐Asp/Cd versus Cd: ##*p* < 0.01; ###*p* < 0.001.

The expression levels of GRP78, a key marker of ER stress, were analyzed. WB results demonstrated that D‐Asp treatment significantly reduced GRP78 expression compared to controls (C) (*p* < 0.05), while Cd exposure led to a pronounced increase in GRP78 expression levels relative to C (*p* < 0.01) (Figure 7A). In the Cd+D‐Asp and D‐Asp/Cd groups, GRP78 expression levels were significantly lower than those in the Cd group (*p* < 0.01) and comparable to control values (Figure 7A).

To further examine the effects of these treatments on ER stress, GRP78 localization was assessed using IF staining (Figure 7B). In the C, D‐Asp, Cd+D‐Asp, and D‐Asp/Cd groups, GRP78 displayed faint localization in the cytoplasm of spermatocytes (striped arrows), round spermatids (solid arrows), and LCs (asterisks; insets), with no significant differences in fluorescent intensity among these groups (Figure 7B,C). However, in the Cd‐treated group, GRP78 signal intensity was markedly elevated, particularly in LCs and spermatids, compared to controls (*p* < 0.01) (Figure 7B,C).

## 4 Discussion

Cd is a pervasive environmental contaminant posing significant risks to human health [4]. Common sources of Cd exposure include dietary intake, cigarette smoke, ambient air, contaminated dust, drinking water, and soil. Numerous studies have demonstrated Cd's toxic effects across various organs, such as the kidney, brain, and testis [14, 28, 29]. These effects are mediated through mechanisms including oxidative stress, inflammation, and cell death [30, 31]. In the testis, Cd inhibits the proliferation of immature SCs, induces DNA damage and cell death, and leads to ultrastructural abnormalities and apoptosis in SCs [32, 33, 34, 35]. Additionally, Cd disrupts the steroidogenesis process in LCs, resulting in reduced serum testosterone (T) levels [9, 11, 36]. This testosterone deficiency impairs mitotic and meiotic progression, affecting spermatogenesis [11]. Despite extensive research, the precise molecular mechanisms underlying Cd‐induced damage to testicular functions remain unclear. Given the critical roles of mitochondria and the ER in steroidogenesis and germ cell maturation, this study examined the effects of Cd on the functionality of these organelles.

In the testes of Cd‐treated rats, increased expression levels of DRP1, a marker of mitochondrial fission, were observed, along with decreased levels of MFN1, MFN2, and OPA1, markers of mitochondrial fusion. These findings align with in vitro studies showing that Cd induces excessive mitochondrial fission in mouse LCs [10]. Additionally, Cd treatment disrupted the mitophagy pathway, as evidenced by elevated PINK1 levels and reduced PARKIN expression. This imbalance suggests that Cd impairs mitophagy, leading to the accumulation of dysfunctional mitochondria, which may release cytochrome c, thereby promoting apoptosis in testicular cells [10]. This mechanism aligns with prior evidence of Cd‐induced apoptosis in rat testes [11]. Further mitochondrial damage was indicated by the downregulation of NRF1 and TFAM, key markers of mitochondrial biogenesis, and decreased expression of TOMM20, a marker of mitochondrial mass. IF analysis confirmed that Cd‐induced mitochondrial damage was most pronounced in LCs, spermatocytes, and spermatids, highlighting the impairment of both steroidogenesis and spermatogenesis caused by Cd accumulation.

Currently, effective therapies to counteract Cd toxicity remain unavailable, generating significant interest in identifying novel therapeutic agents. D‐Asp, an amino acid naturally present in the testis, plays a critical role in male fertility by stimulating testosterone biosynthesis and promoting germ cell proliferation in both in vivo and in vitro models [37, 38, 39, 40, 41, 42]. Previous research has demonstrated that D‐Asp effectively mitigates Cd‐induced toxicity affecting steroidogenesis and spermatogenesis [11]. D‐Asp can counteract/prevent Cd‐induced decrease in serum testosterone levels [11]. Moreover, D‐Asp administration enhances mitochondrial fusion, biogenesis, and the expression of MAM markers in rat testes, as corroborated by the present findings [26, 43].

To investigate the protective role of D‐Asp against Cd‐induced mitochondrial and MAMs destabilization, rats were treated either simultaneously with D‐Asp and Cd for 15 days or with D‐Asp for 15 days prior to Cd exposure for another 15 days. In both protocols, D‐Asp administration fully counteracted Cd's harmful effects on mitochondrial dynamics and function in the testes. Specifically, D‐Asp restored the expression of mitochondrial fusion and fission markers, as well as markers of biogenesis, to levels equal to or exceeding controls. Additionally, both co‐administration and pretreatment with D‐Asp preserved the mitophagy pathway and fully restored TOMM20 expression levels, demonstrating D‐Asp's ability to maintain mitochondrial integrity and functionality under Cd exposure.

The functionality of MAMs, particularly the transfer of lipids between mitochondria and the ER, is a critical step in steroidogenesis and germ cell maturation. Key proteins involved in this lipid transfer include ATAD3A, SOAT1/ACAT1, and FACL4, which facilitate the transport of lipids between mitochondria and ER, playing pivotal roles in steroidogenesis [16, 44]. In the present study, Cd exposure resulted in significantly reduced expression levels of these lipid transfer‐related proteins compared to control rats, indicating disrupted lipid transfer across the intermembrane space. Conversely, D‐Asp treatment notably upregulated the expression of ATAD3A, SOAT1/ACAT1, and FACL4, consistent with previous findings [26]. When D‐Asp was administered either concurrently with Cd or as a pretreatment, it effectively mitigated the adverse effects of Cd on these proteins. In both D‐Asp/Cd and Cd+D‐Asp groups, the expression levels of these markers were significantly higher than those in Cd‐treated rats and even exceeded control levels. This suggests that D‐Asp enhances lipid transfer between mitochondria and ER in germ cells and LCs, thereby maintaining normal testicular function.

The results demonstrated an increase in VDAC and GRP75 expression levels across all experimental groups, particularly in the testis of Cd‐treated rats. VDAC and GRP75 proteins are crucial in facilitating Ca^2+^ transport from the ER to the mitochondria. Elevated expression levels of these proteins may disrupt cellular Ca^2+^ homeostasis [45]. It is hypothesized that this condition in the testis of Cd‐treated rats may be associated with increased oxidative stress and apoptosis, processes widely recognized as being induced by Cd [11]. Consistent with these findings, in vitro exposure of GC‐1 spermatogonia to cadmium chloride (CdCl_2_) has been reported to induce apoptosis through increased ROS levels and Ca^2+^ transfer from the ER to mitochondria, ultimately causing mitochondrial Ca^2+^ overload [11, 46]. In groups treated with D‐Asp (D‐Asp, D‐Asp/Cd, and Cd+D‐Asp), the elevated expression of GRP75 and VDAC may correlate with enhanced steroidogenesis. Notably, mitochondrial Ca^2+^ transfer plays a recognized role in the synthesis of steroid hormones, particularly in steroidogenesis within LCs [47]. However, further investigation is needed to clarify this aspect.

Moreover, the marked upregulation of GRP78 observed in the testis of Cd‐treated rats suggests that this heavy metal induces ER stress. The administration of D‐Asp mitigated this Cd‐induced ER stress, as indicated by reduced GRP78 expression levels in both Cd+D‐Asp and D‐Asp/Cd groups. Thus, it is suggested that the concomitant or preventive administration of D‐Asp may exert protective effects against Cd‐induced testicular damage by improving the oxidative status of the ER.

In conclusion, this study underscores the damaging effects of Cd on mitochondrial compartments and MAMs functionality in the testis, providing insights into the cellular mechanisms of Cd reprotoxicity. Furthermore, the findings highlight the potential role of D‐Asp, commonly used to enhance endogenous testosterone biosynthesis in male infertility treatments, in mitigating Cd‐induced testicular cell damage by strengthening mitochondrial and MAMs functionality.

## Author Contributions

D.L., G.C.B., and M.M.D.F. wrote the main manuscript text. D.L., S.F., M.V., G.G., and I.M. carried out investigations and methodology. D.L. and M.V. prepared the figures. A.S., G.C.B., and M.M.D.F. did supervision, review, and editing. All authors reviewed the manuscript.

## Ethics Statement

The experimental procedure was approved by the Ethics Committee for Research in life science and health of the Higher Institute of Biotechnology of Monastir (CER‐SVS/ISBM—protocol 022/2020) and was carried out according to the UNESCO Recommendation Concerning Science and Scientific Research (1974, 2017).

## Conflicts of Interest

The authors declare no conflicts of interest.

## Data Availability

The data that support the findings of this study are available from the corresponding author upon reasonable request.
